# Mania from Decayed Teeth

**Published:** 1842-12

**Authors:** Wm. Mendenhall

**Affiliations:** Beverly, Anson Co., N. C.


*Mania from Decayed Teeth.* By Dr. WM. MENDENHALL, of Beverly, Anson Co., N. C.—A black boy, about 12 or 14 years of age, was attacked with mania in the month of May, 1840, supposed to have arisen from overheating in trying to subdue a fire which broke out upon his owner's fencing. Cathartics, blistering, cupping and venesection, with many other remedies, were tried, which relieved him, in some measure, during the greater part of the year 1841. But in January, 1842, he became quite a maniac again, and so continued, notwithstanding all the above remedies were used, together with shaving the head and the application of cold water, and opening the temporal artery, and taking considerable blood. A few weeks ago it was discovered that he had two decayed teeth in the upper jaw, one on the right, the canine tooth, the other on the left, one of the anterior molars, both of which were extracted, since which time he has been quite restored. The day before the extraction he escaped from the family, and passed the night in the woods, and was caught in the act of running away, after coming near or to the house in the absence of the family. Since that time he has regularly attended to business without showing any marks of insanity.

*Amer. Journal of Med. Sciences.*

